# Associations of *Toll-Like Receptor* and *β-Defensin* Polymorphisms with Measures of Periodontal Disease (PD) in HIV+ North American Adults: An Exploratory Study

**DOI:** 10.1371/journal.pone.0164075

**Published:** 2016-10-11

**Authors:** Rajeev K. Mehlotra, Noemi B. Hall, Barne Willie, Catherine M. Stein, Aaron Weinberg, Peter A. Zimmerman, Lance T. Vernon

**Affiliations:** 1 Center for Global Health and Diseases, Case Western Reserve University School of Medicine, Cleveland, Ohio, United States of America; 2 Department of Epidemiology and Biostatistics, Case Western Reserve University, Cleveland, Ohio, United States of America; 3 Center for Proteomics and Bioinformatics, Case Western Reserve University School of Medicine, Cleveland, Ohio, United States of America; 4 Department of Biological Sciences, Case Western Reserve University School of Dental Medicine, Cleveland, Ohio, United States of America; 5 Department of Pediatric and Community Dentistry, Case Western Reserve University School of Dental Medicine, Cleveland, Ohio, United States of America; University of Birmingham, UNITED KINGDOM

## Abstract

Polymorphisms in toll-like receptor (*TLR*) and β-defensin (*DEFB*) genes have been recognized as potential genetic factors that can influence susceptibility to and severity of periodontal diseases (PD). However, data regarding associations between these polymorphisms and PD are still scarce in North American populations, and are not available in HIV+ North American populations. In this exploratory study, we analyzed samples from HIV+ adults (n = 115), who received primary HIV care at 3 local outpatient HIV clinics and were monitored for PD status. We genotyped a total of 41 single nucleotide polymorphisms (SNPs) in 8 *TLR* genes and copy number variation (CNV) in *DEFB4*/*103A*. We performed regression analyses for levels of 3 periodontopathogens in subgingival dental plaques (*Porphyromonas gingivalis* [*Pg*], *Treponema denticola* [*Td*], and *Tannerella forsythia* [*Tf*]) and 3 clinical measures of PD (periodontal probing depth [PPD], gingival recession [REC], and bleeding on probing [BOP]). In all subjects combined, 2 SNPs in *TLR1* were significantly associated with *Td*, and one SNP in *TLR2* was significantly associated with BOP. One of the 2 SNPs in *TLR1* was significantly associated with *Td* in Caucasians. In addition, another SNP in *TLR1* and a SNP in *TLR6* were also significantly associated with *Td* and *Pg*, respectively, in Caucasians. All 3 periodontopathogen levels were significantly associated with PPD and BOP, but none was associated with REC. Instrumental variable analysis showed that 8 SNPs in 6 *TLR* genes were significantly associated with the 3 periodontopathogen levels. However, associations between the 3 periodontopathogen levels and PPD or BOP were not driven by associations with these identified SNPs. No association was found between *DEFB4*/*103A* CNV and any periodontopathogen level or clinical measure in all samples, Caucasians, or African Americans. Our exploratory study suggests a role of *TLR* polymorphisms, particularly *TLR1* and *TLR6* polymorphisms, in PD in HIV+ North Americans.

## Introduction

Periodontal diseases (PD) modified by HIV, together with other oral infections, is considered to be among serious complications of HIV infection, and may have implications not only for oral health but possibly for systemic health as well [1–3]. Although the development of PD is generally accepted to depend on the interaction between the host response and the resident oral microbiota [4–6], new vistas into human genomics have opened in an attempt to decipher whether specific genetic polymorphisms can explain host predisposition or susceptibility to PD [6–11]. The components of innate immunity in PD include toll-like receptors (TLRs) [12,13] and β-defensins [14–17]. It is, therefore, not surprising that polymorphisms in these innate immune response genes (*TLR* and *DEFB*, respectively) have been extensively studied as potential genetic factors that may influence susceptibility to and severity of PD [12,18–28]. Although, overall, these studies provide valuable information regarding these genetic associations, they also present some important gaps: First, these studies were conducted primarily in European populations [18–21,26–28]. To the best of our knowledge, data regarding associations between these genetic polymorphisms and PD are still scarce in North American populations [22,24], and are not available in HIV+ North American populations. Second, TLR1 and TLR6, singly and as TLR1/2 and TLR2/6 heterodimers, seem to play a role in PD [29–32]. However, single nucleotide polymorphisms (SNPs) in *TLR1* and *TLR6* were not included in these studies. Third, regarding genetic variation in *DEFB* and its association with PD, while most studies [24,25,27] focused on specific SNPs in *DEFB1*, encoding human β-defensin 1 (hBD-1), only one study [20] focused on copy number variation (CNV) in *DEFB4*, encoding hBD-2. Finally, previous studies have focused on polymorphisms in either *TLR* [18,19,21–23,26,28] or *DEFB* [20,24,25,27] genes. Here, it is important to mention that TLRs have been shown to mediate the expression of hBDs in various tissues [33], and, therefore, the TLR-hBD interplay may be one of the critical determinants of HIV-associated oral infections. TLR2 and TLR4 are involved in hBD-2 induction [34–37] and TLR9 may also be involved [38]. TLR2 is also involved in hBD-3 induction [39], and hBD-3 has been shown to regulate myeloid cell activity through interaction with TLR1 and TLR2 [40–42]. Since myeloid cells play an important role in host immune responsiveness in chronic stages of PD, a full understanding of the interplay between TLRs and hBDs is crucial. Moreover, investigating the cumulative effect of polymorphisms in *TLR* and *DEFB* on PD will provide a more comprehensive understanding of how these interrelated innate immune components influence the complex outcomes in PD and, maybe, other inflammatory-mediated diseases. The present study takes the first step toward closing these gaps.

Previously, to provide a comprehensive view of PD in the era of highly active antiretroviral therapy (HAART), we characterized an urban, predominantly African American HIV+ cohort according to select immunologic and virologic markers, the presence of subgingival pathogens, dental care utilization, and oral health behaviors [43]. We evaluated PD by using distinct clinical measures, i.e., periodontal probing depth (PPD), gingival recession (REC), clinical attachment level (CAL, i.e., PPD+REC), and bleeding on probing (BOP) [2,43]. In addition, we collected subgingival dental plaque samples and quantified DNA levels of specific pathogens associated with severe PD, i.e., *Porphyromonas gingivalis* (*Pg*), *Treponema denticola* (*Td*), and *Tannerella forsythia* (*Tf*) [43]. Thus, our study provided a detailed and extensive characterization of PD that includes the immunological framework of the cohort [44]. To minimize the potential misclassification from using categories (i.e., mild, moderate, and severe; or chronic vs. aggressive), we characterized the distinct clinical measures of PD as continuous variables. This methodology has allowed us to explore the relationship between HIV and PD more comprehensively [2,43,45]. A potential limitation of our study was that the inclusion of a comparison group was not feasible. However, the focus of our original study was to address the interrelationships among clinical, microbial, and immunological factors, and how they are relevant to studying PD in HIV+ adult subjects. It should be noted that this in-depth, singular focus resulted in important epidemiological and methodological findings that can help frame future HIV-related studies in the post-HAART era [44].

**Fig 1:**
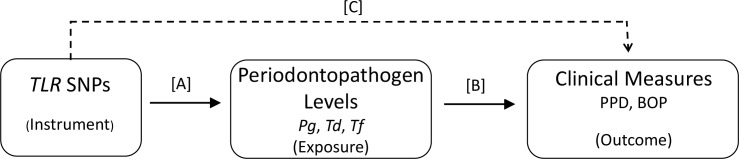
Diagrammatic Representation of Instrumental Variable Analysis. Instrumental variable models use associations C and A to estimate the relationship between an exposure/risk factor and an outcome (B). Note that the instrument is not supposed to have a direct effect on the outcome, hence this line (C) is dashed. Abbreviations: *Pg*, *Porphyromonas gingivalis*; *Td*, *Treponema denticola*; *Tf*, *Tannerella forsythia*; PPD, periodontal probing depth; BOP, bleeding on probing.

The aim of our present exploratory study was to analyze associations between genetic polymorphisms in both *TLR* and *DEFB* genes and periodontopathogen levels and clinical measures of PD in our well-characterized HIV+ cohort [2,43,45]. A long-term goal is to provide an understanding of the biological role of TLR-hBD in PD in HIV+ individuals. For our aim, we evaluated a total of 41 SNPs in 8 *TLR* genes (*TLR1*, *2*, *3*, *4*, *6*, *7*, *8*, and *9*) [46] and CNV in *DEFB4*/*103A*, encoding hBD-2/hBD-3. In addition to linear regression, frequently presented as an optimal method in genetic association studies [47], we employed instrumental variable (IV) analysis [48–50] as a complementary, not comparative, analytic approach. In this approach, a genetic variant is treated as an instrument that is assumed to be associated with an outcome only through its association with an exposure (or risk factor or intermediate variable) (Fig 1). This instrument can then be exploited to obtain causal inferences about the effect of an exposure on an outcome [49,50].

We found that a total of 5 SNPs in *TLR1*, *TLR2*, and *TLR6* were significantly associated with 2 periodontopathogen levels and one clinical measure of PD.

## Materials and Methods

### Study design and participants

In our retrospective study, adult subjects receiving primary HIV care from 3 outpatient HIV clinics in Cleveland, OH, were recruited and monitored for PD status in a research study conducted between May 2005 and March 2009 [2]. Subject recruitment process has been described elsewhere [43,45]. Inclusion criteria were: medication-compliant adult subjects, age 18 or older, who were taking HAART for < 2 years at baseline. Exclusion criteria included evidence of a history of cardiovascular disease or Type I or II diabetes mellitus, fewer than 20 teeth, uncontrolled systemic illnesses, diagnosis or treatment of cancer in the past 5 years, pregnancy, and need for antibiotic prophylaxis prior to dental care as per the American Dental Association and other guidelines [43,45]. IRB approval was obtained from University Hospitals Case Medical Center (UHCMC); all subjects signed a written UHCMC IRB-approved informed consent document.

### Periodontal disease measurements and definition

PPD, REC, and BOP were determined at 6 sites/tooth by one dentist (LTV) as previously described [2,43]. Consistent with our previous report, PD was defined as the percent of teeth with ≥ 1 of 6 sites meeting or exceeding the following thresholds: PPD ≥ 5 mm and REC ≥ 0 mm [43]. BOP was defined as the percent of teeth with ≥ 4 of 6 sites exhibiting BOP [2]. We did not include CAL as a variable into our analyses, because we were unable to process this data for 28% (32/115) of our subjects due to a lack of data management and budgetary resources. We acknowledge that classification of PD has proven to be problematic. PD is challenging to define clinically, and all classification systems produced to date have their imperfections and their critics [51,52]. The use of different case definitions has a great impact on the prevalence and extent rates of PD [53,54], and thus can influence the results and associations presented in studies, including our own. We also collected and pooled subgingival dental plaque samples from 8 pre-determined sites, based on the study by Fleiss et al. [55], and then quantified DNA levels of *Pg*, *Td*, and *Tf* by real-time PCR in units of log genome copy number/μg total DNA as previously described [43].

### Genetic analysis

DNA was extracted from 200 μl of packed blood pellets from study subjects using the QIAamp 96 spin blood kit (QIAGEN, Valencia, CA, USA). A total of 41 SNPs in 8 *TLR* genes (*TLR1*, *2*, *3*, *4*, *6*, *7*, *8* and *9*) were genotyped using Illumina’s GoldenGate genotyping assay system combined with VeraCode Technology (Illumina Inc., San Diego, CA, USA). These SNPs were located in promoter regions, 5′-untranslated regions (UTR), exons, introns, and 3′-UTR (S1 Table). A detailed description of how these SNPs were selected has been provided elsewhere [46]. Allelic discrimination was performed using a BeadXpress Reader (Illumina Inc., San Diego, CA, USA) according to the manufacturer’s instructions.

For the determination of *DEFB4*/*DEFB103A* CNV, the real-time quantitative PCR assay was used as described [56]. Reference genes *TBP* (TATA-Box Binding Protein, GenBank accession #AL031259) and *DEFB1* (encoding hBD-1, GenBank accession #NT_023736), specific primer sets producing only one specific product of ~150 bp at 54°C annealing temperature, reaction mix, and conditions were used as described [56], and the Bio-Rad CFX96TM system (Bio-Rad Laboratories, Hercules, CA, USA) was used for the PCR analysis. Each sample was run in triplicate. Data were analyzed by the comparative Ct method, and the copy numbers were calculated as described [56]. We have assessed the reliability and validity of this assay using 151 multi-population samples, which included 46 well-characterized samples from 5 diverse populations from the Coriell Cell Repositories [57].

### Statistical analysis

Minor allele frequencies (MAF) were calculated using PLINK v1.07. Pairwise linkage disequilibrium (LD) between SNPs of a *TLR* gene or 2 genes that are nearby (*TLR1* and *TLR6* [12 kb], *TLR7* and *TLR8* [10 kb]) was determined for both Caucasians and African Americans using SHEsis (http://analysis.bio-x.cn/myAnalysis.php). Strong LD was defined by high values for both *D*′ (≥0.8) and *r*^2^ (≥0.5) parameters [58].

Linear regression analysis was performed on all 41 SNPs for each of the 3 periodontopathogen levels (*Pg*, *Td*, *Tf*) and the 3 clinical measures (PPD, REC, BOP) using PLINK v1.07. All covariates included in the initial iteration of the regression models are shown in Table 1. Backward stepwise regression was used when selecting significant covariates for each model. Initially, all subjects were included in a single analysis, adjusting for self-identified race. Regression analysis was repeated after stratifying for race, analyzing Caucasians and African Americans separately. SNPs were coded under an additive genetic model, and then under a dominant genetic model [46]. For all SNP association tests, the significance threshold α was determined by using SNPSpDlite [59]. SNPSpDlite calculates a multiple testing correction for SNPs that are in LD with one another, by calculating the LD correlation matrix for given SNPs, then estimating the number of independent tests within the sample. This is an alternative to the more conservative Bonferroni correction, which assumes all tests are independent. Thus, the significance threshold, α, for all SNP association tests was 0.001 (effective number of independent tests = 35). The additive and dominant models were tested separately, with the same significance threshold (0.001) applied to both sets of results.

**Table 1:** Characteristics of the Study Cohort*.

Characteristic	n (%)	Mean (SD)
Age (years)^†^		41 (9.6)
Race^†^		
Caucasian	38 (33%)	
African American	69 (60%)	
Other/Not known	8 (7%)	
Gender^†^		
Male	88 (77%)	
Female	27 (23%)	
Education^†^		
High School/GED or more	55 (48%)	
Smoking^†^		
Ever smoked	73 (64%)	
Number of years smoked		14 (12.0)
BMI (kg/m^2^)^†^		27 (7.5)
History of HTN^†^	31 (27%)	
HIV clinical measures		
Baseline CD4+ T-cell count (cells/μl)		504.6 (324.1)
Baseline viral load (copies/ml)^†^		16519 (48717)
Nadir CD4+ T-cell count (cells/μl)^†^		159 (133.0)
Time since first seropositive (months)^†^		108.2 (83.6)
HAART duration (months)^†^		52.5 (55.1)
Periodontal measures		
PPD ≥ 5 mm (% teeth ≥ 1 site/tooth)		36.1 (24.3)
REC > 0 mm (% teeth ≥ 1 site/tooth)		55.4 (31.1)
BOP ≥ 4 sites/tooth (% teeth)		47.4 (20.1)
Microbial measures		
(log genome copy number/μg DNA)		
*Porphyromonas gingivalis*		3.69 (2.6)
*Treponema denticola*		3.93 (2.3)
*Tannerella forsythia*		4.77 (2.1)

*Abbreviations: GED, general educational development; BMI, body mass index; HTN, hypertension; HAART, highly active antiretroviral therapy; PPD, periodontal probing depth; REC, gingival recession; BOP, bleeding on probing.

^†^Covariates included in the initial iteration of the regression models.

To determine whether a statistically significant relationship existed between a periodontopathogen level and a clinical measure, preliminary linear regression analysis on all samples was conducted in R (http://www.r-project.org/). Again, backward stepwise regression was used to select only those covariates which significantly contributed to the model. If a significant relationship (p<0.001) was identified between a given periodontopathogen level and clinical measure, only then was the pair included in the IV analysis.

Instrumental variable analysis was used to test the hypothesis that a given *TLR* SNP may be driving the relationship between each periodontopathogen level and clinical measure pair [60,61]. Each of the 41 SNPs was tested as an IV, while a periodontopathogen level was treated as the exposure and a clinical measure as the outcome. We performed IV analysis using a 2-stage method comprising 2 regression stages: the first-stage regression of the exposure on the IVs, and the second-stage regression of the outcome on the fitted values of the exposure from the first stage. The IV analysis was performed using the ivreg function within the AER package (http://CRAN.R-project.org/package=AER). Two p values were generated: the first p value (p_A_) describes the relationship between the *TLR* SNP (IV) and the periodontopathogen level (exposure), and the second p value (p_B_) describes the mechanistic relationship between the periodontopathogen level (exposure) and clinical measure (outcome) (Fig 1). Both p values <0.05 were considered significant.

To analyze the effect of CNV on each of the 6 measures of PD, linear regression analysis was performed in R. Two different approaches were used to include CNV in the regression model: binary or categorical. Using a cutoff at the median (CNV = 5), CNV was recoded as a binary variable where 1 = CNV ≥ 5, 0 = CNV < 5 (reference group). To create the categorical variable, CNV was divided into CNV < 5, CNV = 5 (reference group), and CNV > 5. For each model, binary or categorical, backward stepwise regression was used as before to select only those covariates which significantly contributed to the model at the α = 0.05 level.

## Results

### Study subjects, minor allele frequencies, and linkage disequilibrium patterns

The cohort characteristics, including demographics, HIV infection correlates, and PD measurements of the study subjects (n = 115), are presented in Table 1. The majority of the subjects were African Americans, with a predominance of males. The MAF of all 41 *TLR* SNPs in all subjects combined are presented in S2 Table. MAF ranged from 0.01 to 0.50, which concurred with those reported by our group for another HIV cohort, with similar race and gender distribution, from the same geographic area [46]. The pairwise strong LD patterns of *TLR* genes for both Caucasians and African Americans are presented in S3 Table. Caucasians showed a higher overall extent of LD than African Americans.

### Regression analysis of SNPs

In all subjects combined, 2 SNPs in *TLR1* (−2192T>C, 1805G>T) were significantly associated with *Td* (Table 2), and one SNP in *TLR2* (597T>C) was significantly associated with BOP (Table 3). One of the 2 SNPs in *TLR1* (1805G>T) was significantly associated with *Td* in Caucasians (Table 2). In addition, another SNP in *TLR1* (−7202G>A) and a SNP in *TLR6* (−502T>C) were also significantly associated with *Td* and *Pg*, respectively, in Caucasians (Table 2).

**Table 2:** Association Between Periodontopathogen Levels and *TLR* SNPs*.

Group	Periodonto-pathogen	Gene	rs number	SNP	Amino acid	Allele	Test	β	*p* value
All samples	*Td*	*TLR1*	rs5743595	−2192T>C	-	C	Add	1.27	0.001^a^
							Dom	1.80	0.001^a^
			rs5743551	−7202G>A	-	A	Add	-0.95	0.002
			rs5743618	1805G>T	Ser602Ile	G	Add	-1.11	0.001^a^
		*TLR6*	rs5743795	−1401G>A	-	A	Add	1.39	0.004
							Dom	1.48	0.004
Caucasian	*Pg*	*TLR6*	rs1039559	−502T>C	-	C	Add	-2.10	0.001^a^
	*Td*	*TLR1*	rs5743551	−7202G>A	-	A	Add	-1.57	0.001^a^
			rs5743618	1805G>T	Ser602Ile	G	Add	-1.56	<0.001^a^
African American	*Td*	*TLR1*	rs5743618	1805G>T	Ser602Ile	G	Add	-1.72	0.009
		*TLR2*	rs4696480	−16934T>A	-	T	Dom	-1.37	0.006
		*TLR6*	rs5743810	745T>C	Ser249Pro	T	Add	-1.81	0.003
		*TLR8*	rs5744077	28A>G	Met10Val	G	Add	-1.74	0.003
			rs5744080	645C>T	His215His	C	Add	-1.37	0.008
	*Tf*	*TLR1*	rs5743618	1805G>T	Ser602Ile	G	Dom	-1.51	0.002
		*TLR8*	rs1548731	+3121T>C	-	T	Add	0.86	0.004
		* *	rs5744077	28A>G	Met10Val	G	Add	-1.22	0.008

*Abbreviations: *Td*, *Treponema denticola*; *Pg*, *Porphyromonas gingivalis*; *Tf*, *Tannerella forsythia*; Add, additive genetic model; Dom, dominant genetic model.

^a^Significant after the correction for multiple testing (α = 0.001).

**Table 3:** Association Between Clinical Measures of PD and *TLR* SNPs*.

Group	Clinical measure	Gene	rs number	SNP	Amino acid	Allele	Test	β	*p* value
All samples	REC	*TLR6*	rs2381289	4224C>T	-	T	Dom	13.26	0.007
	BOP	*TLR2*	rs3804099	597T>C	Asn199Asn	T	Add	8.89	0.001^a^
							Dom	14.66	<0.001^a^
		*TLR9*	rs187084	−1486C>T	-	C	Add	-7.31	0.010
Caucasian	PPD	*TLR4*	rs10759932	−1607T>C	-	C	Dom	18.12	0.005
	REC	*TLR2*	rs1898830	−15607A>G	-	G	Add	12.92	0.006
							Dom	15.87	0.006
	BOP	*TLR9*	rs352139	+1174G>A	-	A	Dom	20.57	0.004
			rs352140	1635G>A	Pro545Pro	A	Add	-12.98	0.006
African American	REC	*TLR6*	rs2381289	4224C>T	-	T	Dom	19.66	0.005
	BOP	*TLR2*	rs3804099	597T>C	Asn199Asn	T	Dom	14.70	0.004

*Abbreviations: REC, gingival recession; PPD, periodontal probing depth; BOP, bleeding on probing; Add, additive genetic model; Dom, dominant genetic model.

^a^Significant after the correction for multiple testing (α = 0.001).

In addition to the aforementioned *TLR* SNPs, which were significant after the correction for multiple testing (α = 0.001), there were SNPs that were associated with the periodontopathogen levels (Table 2) and clinical measures (Table 3) at a lower significance level of 0.002–0.01. These p values may be indicative of suggestive associations, which require further validation and/or larger sample sizes. Comparing the results presented in Table 2 and Table 3, no *TLR* SNP was common to both periodontopathogen levels and clinical measures. The *TLR2* SNPs were not in LD, and the *TLR6* SNP LD patterns differed between Caucasians and African Americans (S3 Table).

### Instrumental variable analysis

Preliminary linear regression analysis on all samples showed that all 3 periodontopathogen levels were significantly associated with PPD and BOP (p<0.001), but none was associated with REC (p≥0.02) (S4 Table).

Instrumental variable analysis using all 41 *TLR* SNPs (IVs), the 3 periodontopathogen levels (exposures), and PPD and BOP (outcomes) showed that 8 SNPs in *TLR1* (n = 2), *TLR2* (n = 1), *TLR4* (n = 1), *TLR6* (n = 1), *TLR8* (n = 2), and *TLR9* (n = 1) were significantly associated with the 3 periodontopathogen levels (*Pg*, 2 SNPs; *Td*, 6 SNPs; *Tf*, 1 SNP) (p_A_<0.05) (Fig 1, S5 Table). These p_A_ values suggest that there is a relationship between these 8 IVs and 3 exposures. However, none of the periodontopathogen levels were associated with PPD or BOP after accounting for the effects of these SNPs (p_B_≥0.1) (Fig 1, S5 Table). These p_B_ values suggest that the relationship between the 3 exposures and 2 outcomes is not driven by these IVs.

### Distribution and association of CNV

**Fig 2:**
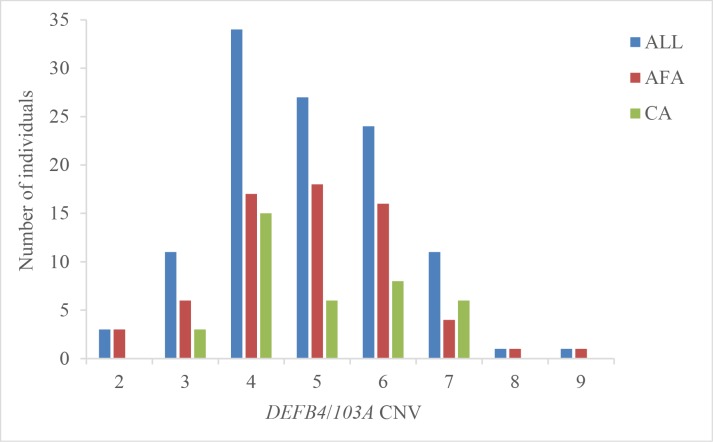
Distribution of *DEFB4*/*103A* CNV in All Samples, African Americans (AFA), and Caucasians (CA).

The distribution of integer *DEFB4*/*103A* copy numbers in all samples and the 2 racial groups is shown in Fig 2. No difference was observed in the median and mean copy numbers in all samples (5.0 and 4.9, respectively), Caucasians (5.0 and 5.0, respectively), and African Americans (5.0 and 4.9, respectively). Linear regression analysis using a binary (CNV ≥ 5, CNV < 5) or categorical (CNV < 5, CNV = 5, CNV > 5) model did not show any association between CNV and any periodontopathogen level or clinical measure in all samples, Caucasians, or African Americans (p>0.05, data not shown).

## Discussion

In this exploratory study, utilizing samples from HIV+ North American subjects, we found that a total of 5 SNPs in *TLR1* (n = 3), *TLR2* (n = 1), and *TLR6* (n = 1) were significantly associated with 2 periodontopathogen levels (Table 2) and one clinical measure of PD (Table 3).

TLR1 gene and/or protein expression may be upregulated in PD [29,32], and TLR1/2 heterodimer signaling may be involved in inflammatory response in PD [30,31]. To date, no report is available regarding the role of *TLR1* SNPs in PD. We found that *TLR1* −2192C, −7202A, and 1805G alleles were significantly associated with *Td* (Table 2). The information regarding the functional relevance of −2192T>C is limited. The −2192C allele may be protective for atopic asthma [62]. In that report, PBMCs of atopic asthma patients carrying −2192C showed increased expression of TLR1 mRNA and protein, increased production of proinflammatory cytokine TNF-α and T_H_1 cytokines IL-12 and IFN-γ, and decreased production of T_H_2 cytokine IL-4 after stimulation with TLR1/2 ligand Pam3CSK4 [62]. −7202G>A and 1805G>T were functionally relevant in sepsis, tuberculosis, leprosy, and candidemia, where −7202A and 1805G were associated with lower NF-κB activation and signaling, and decreased inflammatory cytokine production, including that of IL-6 (references cited in [46]). The lipooligosaccharide of *Td* has been shown to bind to gingival fibroblasts, and this binding is mediated by the co-receptor CD14 [63]. Furthermore, stimulating fibroblasts with *Td* lipooligosaccharide significantly increased the secretion of IL-6 [63]. CD14 and IL-6 are known to be involved in the inflammatory process of PD [64,65]. Thus, our finding that *TLR1* −2192C, −7202A, and 1805G alleles are significantly associated with *Td* is noteworthy, and may be considered as a starting point in identifying the contribution of *TLR1* variation to PD.

TLR2 gene and/or protein expression may be upregulated [12,29,32], not affected [66], or downregulated in PD [67]. Soluble salivary TLR2 may be considered a potential prognostic or periodontal health maintenance marker for chronic periodontitis [64]. We found that *TLR2* 597T allele was associated with BOP (all samples, p≤0.001; African Americans, p = 0.004) (Table 3). BOP has long been considered an indicator of a subgingival inflammatory response to bacterial pathogens; there was a direct relationship between BOP and subgingival endoscopic biofilm and calculus indexes [68]. In our study, the levels of *Pg*, *Td*, and *Tf* DNA in subgingival plaque were highly correlated with BOP (S4 Table). Others have reported that the mRNA levels of *TLR2* in human gingival fibroblasts were positively correlated with the number of *Pg* in subgingival plaque [69]. This *Pg*-induced *TLR2* expression may be partially dependent on TNF-α [69].

Most previous studies did not include 597T>C [12,19,22,23]. One study, conducted in 2 Northwest European populations, did include this SNP [21] but found no association with PD. Being a synonymous SNP (Asn199Asn), the molecular mechanism of 597T>C is not clear. The 597T allele may have a role in protection against *Mycobacterium tuberculosis* (Mtb), *M*. *leprae* [70,71], and the lymphatic filariasis nematode *Wuchereria bancrofti* [72], which harbors an intracellular symbiotic bacterium *Wolbachia*. It may be that 597T>C is in LD with a highly polymorphic (GT)n dinucleotide repeat within intron-2 that affects gene regulation [71]. However, the (GT)n repeat polymorphism was not previously associated with PD [18]. In addition, 597T>C is in LD with a 22-bp insertion-deletion polymorphism (Δ22 [−196 to −174]) in the 5′-UTR [72]. Located in close proximity to the NF-κB and SP1 transcription factor binding sites, *TLR2* Δ22 is a functional polymorphism [72]. We did not study the (GT)n repeat and Δ22 polymorphisms, but they could form the basis of a future study. Given that HIV/AIDS continues to disproportionately affect African Americans, evaluating the functional and clinical effects of 597T>C in further studies is highly relevant.

TLR6 protein may be upregulated in PD [29], and TLR2/6 heterodimer signaling may be involved in inflammatory responses in PD [31]. However, as is the case for *TLR1* SNPs, the role of *TLR6* SNPs in PD has not yet been determined. We found that *TLR6* −502C allele was significantly associated with *Pg* in Caucasians (Table 2). The functional relevance of this SNP could be due to the fact that it is in strong LD with non-synonymous 745T>C (−502C-745T) (S3 Table). Others have found 745T protective in tuberculosis patient studies and associated with lower NF-κB signaling, decreased production of IL-6 in whole blood stimulated with TLR2/6 ligand PAM2 and Mtb lysate [73], and increased production of IFN-γ in whole blood stimulated with Bacillus Calmette-Guérin as well as PBMCs stimulated with TLR1/6 lipopeptide ligands [74]. In addition, −502T>C is in strong LD with 4224C>T (S3 Table), located in the 3′-UTR. It is well known that the 3′-UTR significantly determines the stability, localization, translation, and degradation of mRNA [75].

All 3 periodontopathogen levels were significantly associated with PPD and BOP (S4 Table). In our IV analysis, a total of 8 SNPs were significantly associated with the 3 periodontopathogen levels, the majority with *Td* (p_A_ values, S5 Table). However, the relationship between each periodontopathogen level and clinical measure pair was not driven by associations with these identified SNPs (p_B_ values, S5 Table). Instrumental variable analysis is being increasingly employed in epidemiology to investigate the potential causal effects of an exposure [60,61]. However, major limitations of this analysis result from the strict assumptions that need to be satisfied for the method to be reliable [49,50]: Although *TLR* SNPs were associated with the periodontopathogen levels (exposures), they may be “weak instruments”–such an IV explains a relatively small proportion of variance in the exposure. It may also be that there is limited statistical power. Insufficient statistical power is a common characteristic of many IV analysis studies and, therefore, a careful selection of instruments plus an adequate sample size are deemed necessary for this method to be able to make reliable conclusions. It is also possible that pleiotropy, where one genetic variant has multiple functions, may be occurring. Among the 5 significant SNPs in this study, 3 (*TLR1* −7202G>A, 1805G>T; *TLR6* −502T>C) were associated with HIV status in our previous study conducted on another similar HIV cohort [46]. Our study subjects, particularly African Americans, may have population stratification, as the contribution of European ancestry to African-American populations can vary substantially (3% to >30%) [46]. Finally, while we tested for common confounders, it is possible that hidden confounding from unmeasured variables may have affected the analysis. Despite these limitations, 6 of the 8 SNPs from our IV analysis should be investigated further, as they were also significantly or suggestively associated with the periodontopathogen levels or clinical measures of PD (S5 Table).

We did not find an association between *DEFB4*/*103A* CNV and any of the measures of PD in any analysis (data not shown). This result seems contrary to the only report available, which found an association between low *DEFB4* CNV and severe form of periodontitis [20]. There could be a number of reasons for this difference: First, Jaradat et al. [20] analyzed the association between *DEFB4* CNV and generalized chronic periodontitis severity, classified as slight-to-moderate and severe subgroups. We, on the other hand, evaluated PD by its component parts (the 3 selected clinical measures of PD) because they may represent different biological processes contributing to PD [43]. To minimize the possibility of misclassification by categorizing data, we defined and analyzed all PD data as continuous variables. Using this approach, we have uncovered associations between PD measures and markers of cardiovascular disease risk [2] as well as PD measures and HIV-related immunological markers in the era of HAART [45]. Second, using different methods, it has been found that *DEFB4*/*103A* genes consistently vary from 2 to 12 copies per diploid genome, with a median or mean copy number of 4 in most populations [57], including the one analyzed by Jaradat et al. [20]. In our study, the median and mean copy number was 5 in all population groups. This could be related to the relatively small sample size (n = 115)–a factor that may have also contributed to the lack of association. Finally, our study was not designed to measure the hBD-2/hBD-3 protein concentrations.

In conclusion, our exploratory study provides new insights into the association of genetic variation in *TLR* with PD measures in HIV+ North Americans. To the best of our knowledge, this is the first study to analyze the role of *TLR1* and *TLR6* SNPs in PD; therefore, we cannot directly compare our findings to other studies. The mechanisms by which the aforementioned *TLR* SNPs, singly, in haplotypes, or in heterodimers, influence PD need to be further elucidated. Analysis of mRNA and protein levels of the *TLR* variants, and investigation of interactions of the *TLR* variants with adapter molecules and subsequent recruitment of downstream targets are needed to define the biological mechanisms that underlie these genetic associations.

## Supporting Information

S1 Table Characteristics and Distribution of *TLR* Genes and SNPs. (XLSX)

S2 Table *TLR* Minor Alleles and Their Frequencies in All Samples. (XLSX)

S3 Table Pairwise Linkage Disequilibrium Between *TLR* SNPs. (XLSX)

S4 Table Association Between Periodontopathogen Levels and Clinical Measures. (XLSX)

S5 Table Instrumental Variable Analysis. (XLSX)
